# Mullerian Adenosarcoma of Uterus with Sarcomatous Overgrowth and Heterologous Component Associated with Stromal Deposit in Omentum: A Case Report and Review of the Literature

**DOI:** 10.1155/2012/820378

**Published:** 2012-08-16

**Authors:** Anuradha Sinha, Jyoti Prakash Phukan, Sanjay Sengupta, Paulami Guha

**Affiliations:** ^1^Department of Pathology, Bankura Sammilani Medical College, Bankura, West Bengal, India; ^2^Department of Obstetrics and Gynecology, Calcutta Medical College, Kolkata, West Bengal, India

## Abstract

*Background*. Mullerian adenosarcoma with sarcomatous overgrowth (MASO) is a very rare variant of uterine sarcomas first described by Clement et al. as early as 1974. The presence of heterologous sarcomatous components is associated with aggressive biological behavior. *Case Presentation*. This is a case report of a 62-year female (P_2 + 0_) presenting with postmenopausal vaginal bleeding. Her preoperative USG revealed subserosal fibroid with adherent omentum. She underwent abdominal hysterectomy with bilateral oophorectomy. Histopathological diagnosis of resected specimen was Mullerian adenosarcoma with sarcomatous overgrowth and presence of heterologous elements involving body of the uterus. The whole thickness of the myometrium was involved along with the presence of serosal nodules and omental deposits of sarcomatous component. *Conclusion*. MA is considered as a low-grade malignant tumor, but MASO is a high-grade tumor frequently associated with invasion and metastasis with poor treatment outcome. Because of its rarity, correct identification of these tumors and distinction from other uterine sarcomas are a challenging job and hence its morphological features merits attention.

## 1. Introduction

Malignant stromal tumors account for 1–3% of all female genital tract tumors and Mulierian adenosarcomas (MAs) constitute 8–10% of this malignancies [1–3]. Typically, MA involves endometrium and presents as a polypoid mass. But infrequently these malignancy is reported from ovaries, cervix, vagina, peritoneum, and even pouch of Douglas [3–7]. 

MA is a tumor of postmenopausal women and the commonest presentation is vaginal bleeding [7]. According to classical description by Clement and Scully [1], MA is a mixed stromal and epithelial neoplasm. Stromal component shows malignancy but glandular tissue, though at focal areas may present with atypical proliferation and is devoid of malignant features.

Further classification of MA is done depending upon mesenchymal elements; homologous tumors are composed of nonspecific spindle-shaped sarcomatous cells whereas special differentiation of mesenchymal components into cartilage, osteoid, or striated muscles is associated with heterologous neoplasms [8].

Mullerian adenosarcoma with sarcomatous overgrowth (MASO) is a rare variant of MA, characterized by growth of a second pure high-grade sarcoma occupying at least one-fourth of the tumor mass [9]. In contrast to MA, MASO runs a biologically aggressive course [9, 10].

## 2. Case Report

This 62-year female (P_2+0_) came to gynecology OPD with complains of postmenopausal vaginal bleeding. Preoperative ultrasonography of pelvis revealed presence of a subserosal fibroid with omental nodules. She underwent abdominal hysterectomy with bilateral salpingoophorectomy along with excision of omental nodules.

**Figure 1:**
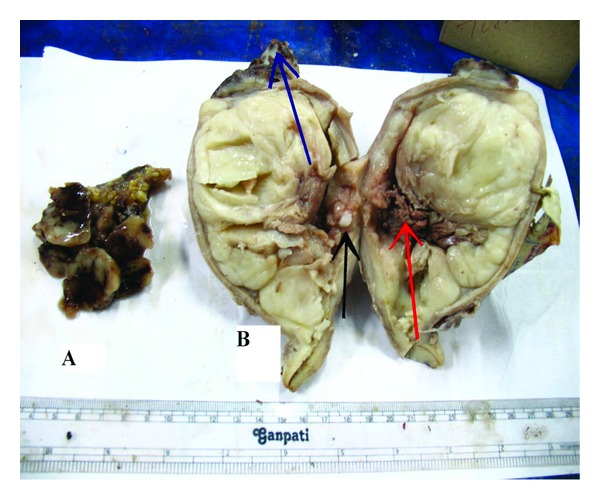
(a) Gross photograph of omental tissue showing haemorrhagic, necrotic fleshy mass. (b) Gross photograph of cut section of uterus showing fleshy mass distorting uterine cavity with invasion of myometrium with areas of haemorrhage and necrosis (Red arrow), bony tissue (black arrow), and subserosal nodule (blue arrow).

**Figure 2:**
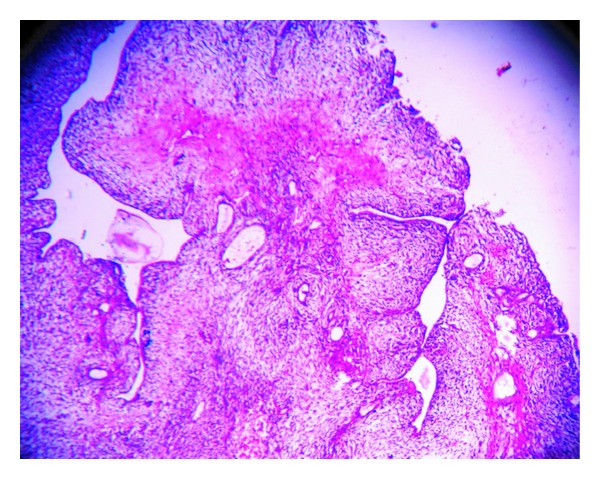
Photomicrograph showing phyllodes-like architecture on low power (H&E stain, ×100).

**Figure 3:**
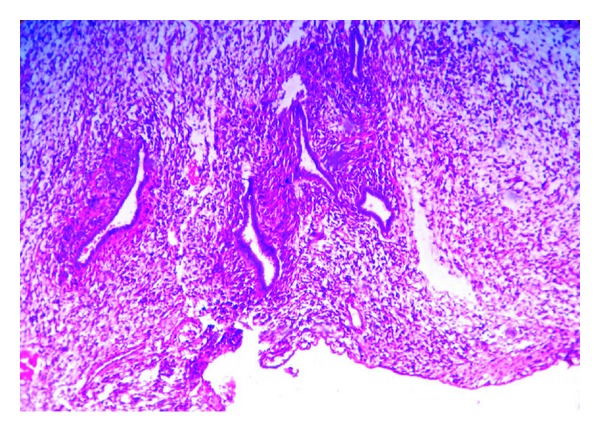
Photomicrograph showing condensation of hypercellular stroma forming periglandular cuffs around glandular slit-like spaces (H&E stain, ×200).

**Figure 4:**
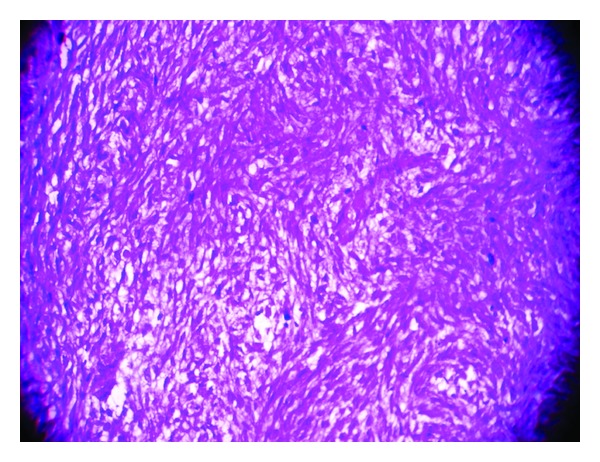
Photomicrograph showing areas of sarcomatous overgrowth having stromal cells displaying obvious cytologic atypia and increased mitotic figures (H&E stain, ×400).

Gross examination showed a fleshy mass distorting uterine cavity with invasion of myometrium as deep as to serosal surface (Figure 1). There was also evidence of necrosis. Tubes, ovaries, and cervix were free of tumor. Omental tissue also showed presence of similar type of haemorrhagic, necrotic fleshy tissue (Figure 1). Histopathological examination of uterine fleshy mass showed presence of dilated and compressed glands lined by benign low cuboidal cells with focal multilayering placed in a cellular stroma composed of plump spindle cells with nuclear atypia (Figures 2 and 3). Sections from different parts of the tumor mass showed a more cellular sarcoma with prominent pleomorphic atypical nuclear features and high rate of mitosis (more than 10/10 high power field) (Figure 4). Microscopic evidence of deep myometrial invasion was also present. At focal areas, bony tissue, malignant cartilage, and skeletal muscle tissue were seen. Omental tissue was also involved by high-grade spindle cell sarcoma. Cervix, tubes, and ovaries were free of tumor.

Histopathological final diagnosis was heterologous mullerian adenosarcoma with sarcomatous overgrowth showing deep myometrial invasion and associated with omental deposit of sarcomatous component.

## 3. Discussion

MA can affect women of any age but reported median age (58 years) is quite close to the age of the present case. Apart from commonest clinical presentation of bleeding per vagina, as also experienced in our patient, other usual presenting features include enlarged uterus, pelvic mass, recurrent uterine polyp, or a mass protruding through external os. These neoplasms usually present as solitary polypoidal endometrial masses. Myometrial invasion was observed infrequently. Hyperestrogenemia, chemotherapy, or radiotherapy may predispose to tumor formation [11–13].

Usual variants of MA under low power light microscopy show a leaf-like architectural pattern lined by bland appearing epithelium resembling phyllodes tumor of the breast. There is stromal condensation around glandular components (mimicking cambium layer) exhibiting maximum cellular atypia and mitotic activity. Other parts are composed of moderately cellular spindle cell stroma with mild-to-moderate cellular atypia and a mitotic count >2/10 high power fields. Heterologous components like skeletal muscle, cartilage, and so forth may be present. Sarcomatous overgrowth in mullerian adenosarcoma is characterized by >25% high-grade stromal component. Nuclear atypia is marked with increased mitotic count (often more than 10/10 high power fields). Stromal cells of classical MA show immunoreactivity for estrogen and progesterone receptors (ER and PR) and CD-10. Loss of expression of hormone receptors and CD-10 is associated with sarcomatous overgrowth.

Differential diagnosis of adenosarcoma of uterus includes adenofibroma, carcinosarcoma, low-grade endometrial stromal sarcoma with glandular differentiation, endometrial polyp, and atypical polypoid adenomyoma (APA). Adenofibroma is characterized by benignity of both epithelial and stromal components without stromal atypia and mitosis. Carcinosarcomas are relatively common tumors without leaf-like arrangement or cambium layer pattern growth. Epithelial component is malignant and mesenchymal component is comparatively higher-grade sarcoma. Endometrial stromal sarcoma may contain focal benign endometrial glands, but irregular distribution of glands and lack of periglandular cuffing differentiate it from adenosarcoma. APA is difficult to differentiate from MA because stromal atypia and focal condensation are often present. Extensive squamous metaplasia and fibromyxomatous stroma favor diagnosis of APA though close follow up is mandatory [11, 14, 15]. 

Adenosarcomas are considered as a low-grade malignant neoplasm with reported local recurrence in 25% cases [12]. The most important histologic poor prognostic indicators are sarcomatous overgrowth and deep myometrial invasion [11]. Other unfavourable prognostic factors include presence of heterologous elements, necrosis, high mitotic rate, and extrauterine spread [9, 10, 12]. Vascular invasion, rarely seen, represents a poorer prognosis [11, 16]. Overall metastasis is reported in 5% of cases with the metastatic component solely represented by the sarcomatous element, consistent with findings of the present case [11, 12]. Median survival time of patients with uterine MASO is 13 months [12, 17].

In this paper, we are presenting a rare uterine tumor with uncommon histological features. Involvement of whole thickness of myometrium with formation of serosal nodules and omental deposits is a rare presentation of MASO. Treatment outcome in this particular case was poor because of association of multiple unfavourable prognostic factors like sarcomatous overgrowth, deep myometrial invasion, high mitotic index, necrosis, presence of heterologous elements, and extrauterine spread. However, MASO should be taken into differential diagnosis of all high-grade uterine sarcomas and multiple sections must be examined to rule out presence of benign glandular element with periglandular cuffing of stromal component.
